# Editorial: Epigenetic regulation behind plant-microbe interactions

**DOI:** 10.3389/fpls.2024.1385356

**Published:** 2024-02-28

**Authors:** Juan Ignacio Vílchez, Serena Varotto, Ho Won Jung

**Affiliations:** ^1^ iPlantMicro Lab, Instituto de Tecnologia Química e Biológica (ITQB)-NOVA, Lisboa, Portugal; ^2^ Department of Agronomy, Food, Natural Resources, Animals and Environment (DAFNAE), Università degli Studi di Padova, Legnaro, Italy; ^3^ Department of Molecular Genetics, Dong-A University, Busan, Republic of Korea

**Keywords:** epigenetics, plant-microbe associations, DNA methylation, hyper/hypomethylated regions, bisulfite sequencing (BS-seq)

Plant responses to their environment involve intricate and diverse genomic regulations at various levels. These responses can be modulated by the epigenetic status of different mechanisms. In recent years, numerous studies have linked DNA/RNA methylation [as linked gene 5-methylcytosine (^5m^C)], demethylation, and chromatin remodeling through acetylation, methylation, or ubiquitination with changes in plant responses to the diverse environment conditions. In this sense, interactions with microorganisms are one of the most significant triggers of plant responses (Chen et al., 2022). Previous research has reported significant alterations in the composition of plant-associated microbiomes when certain epigenetic regulatory mechanisms in plants are activated or repressed (Kaushal et al., 2021). These mechanisms can affect the timing of plant defense systems after pathogen infection and the recruitment of beneficial microorganisms from the environment to combat environmental stresses, among others. Notably, some of these mechanisms have been reported to prime responses for interactions and anticipate robust responses to similar situations in the future. Furthermore, these mechanisms can be inherited by subsequent generations through the transmission of modification patterns. Similarly, microorganisms also employ specific epigenetic regulatory mechanisms to interact with plants. The aim of this Research Topic was to investigate and compile the most recent approaches to epigenetic regulation within the context of plant-microorganism interactions.

The studies included in this Research Topic demonstrate the significance of evaluating the methylation patterns as a key approach for assessing epigenetic regulation in plant-microbe interactions (Chen et al., 2022). Agius et al. highlighted through a review the importance of examining DNA methylation in relation to growth, development, stress response, and adaptability in various plant species. By understanding the methylation status of plants in response to different events, researchers can develop strategies to enhance their productivity and stress tolerance. A range of techniques, such as bisulfite sequencing, methylation-sensitive amplified polymorphism, genome-wide DNA methylation analysis, methylated DNA immunoprecipitation sequencing, and reduced representation bisulfite sequencing, can serve as both a starting point and a multidisciplinary methodology for evaluating the impact of interactions on methylome regulation. However, the success of the analysis ultimately depends on a comprehensive understanding of each technique and its appropriate application. In cases where microbial interactions affect other aspects such as chromatin remodeling, which may be reflected in the methylome, different approaches and methodologies may be required (Zhu et al., 2016).

The investigation of defensive mechanisms against pathogens employed by plants in the field of epigenetics is among the most intriguing research areas. Here, the employment of mutants that pertain to epigenetic regulation has demonstrated to be a highly effective means of elucidating the underlying mechanisms of plant-microorganism interactions. Previous studies have utilized these mutants to investigate modifications in root exudates and the selective beneficial bacteria recruitment processes in *repressor of silencing 1* (*ros1*) mutants (Vílchez et al., 2020), as well as the role of *increase in bonsai methylation 1* (*ibm1*) mutants in assessing autoimmunity and shaping the root microbiome (Lv et al., 2022). In their study, Sun et al. used lesion mimic mutants as the primary technique to better understand plant-microbe interactions and the immune response of plants. They utilized the *lesion mimic phenotype1-1* (*lmp1-1*) mutant to demonstrate that the defense mechanism was activated, particularly through the activation of the phenylalanine ammonia lyase (PAL) pathway, which resulted in the accumulation of salicylic acid (SA). This gene is related to H2B deubiquitination mechanisms, which are thought to regulate the immune response by modifying the SA biosynthesis pathway in rice. This research provides a novel approach to understanding the mechanisms of the plant immune response to pathogenic microorganisms.

Another method, in this case for exploring mutualistic plant-microorganism interactions, was presented by Forte et al. in this Research Topic. This study utilized reduced representation bisulfite sequencing (epiGBS) to examine the influence of the mutualistic strain *Epichloë* sp. LpTG-3 strain AR37 on the methylome of *Lolium perenne*. Results indicated that the presence of this fungal strain led to decreased DNA methylation, a phenomenon known as hypomethylation, across various genomic features. This hypomethylation was consistent in grass species across generations. This observation suggests that factors such as the duration of the interaction between host and mutualist, and the accumulation of genetic and epigenetic changes over time may be linked to the host plant’s genome hypomethylation.

There are also reported different cases beyond the study of the impact of bacteria or fungi on epigenetic regulation in plants, as in the case of the study by Tselika et al. These authors conducted a study in which they analyzed plants infected with viroids and assessed RNA-directed DNA methylation (RdDM) to understand the origin of certain phenotypes in plants. They used *Nicotiana benthamiana* as model plant and discovered that the potato spindle tuber viroid (PSTVd) allowed them to identify endogenous gene promoters and transposable elements targeted by 24 nt host siRNAs that differentially accumulated in PSTVd-infected and healthy plants. These targets were evaluated for their methylation status using digested genomic material with methylation-sensitive restriction enzymes coupled to a polymerase chain reaction (PCR), in this case CHOP-PCR. These methods are critical in studies of this type and allow for the evaluation of an additional layer of complexity in plant-microorganism regulation. Babu and collaborators also found effects on methylation caused by viruses through this approach (Babu et al., 2018). In addition, the authors supported their research with Methylation Sensitive Amplification Polymorphism (MSAP) followed by sequencing (MSAP-seq) to examine genomic DNA methylation of 5-methylcytosine (^5m^C) in CG sites upon viroid infection (complemented with bisulfite sequencing). This allowed them to obtain a high-resolution view of host epigenetic regulation under this type of interaction and identify several target loci differentially methylated upon PSTVd infection.

The kind of works we mentioned above highlights the need for an improved resolution of results when several approaches are included in the design. In their collaboration on this Research Topic, Jahed and Hirst investigated this the multidisciplinary approach to evaluate the epigenetic regulation in fruit growth and development. The intricate regulatory mechanism that governs fruit growth involves a series of events that occur over a growing season, making it imperative to approach the study from a multidisciplinary perspective and employ an analysis methodology that accounts for its unique characteristics. To address this challenge, the authors advocated for the use of high-throughput sequencing technology as a foundation and combining it with the resolution provided by techniques such as phenotyping. By adopting this approach, several studies in the past decade have successfully established direct connections between epigenetic regulation and fruit size, as well as uncovered evolutionary aspects of related domestication processes. Although this method allows for greater resolution in discovering new mechanisms of plant-microorganism interactions, the authors emphasized that is crucial to develop statistical and computational methods to enhance the analysis capacity, taking into account the complexity of each strategy and methodology involved.

## Author contributions

JIV: Conceptualization, Formal analysis, Funding acquisition, Resources, Supervision, Validation, Visualization, Writing – original draft, Writing – review & editing. SV: Conceptualization, Writing – review & editing. HJ: Conceptualization, Writing – review & editing.
